# Interpreting models interpreting brain dynamics

**DOI:** 10.1038/s41598-022-15539-2

**Published:** 2022-07-21

**Authors:** Md. Mahfuzur Rahman, Usman Mahmood, Noah Lewis, Harshvardhan Gazula, Alex Fedorov, Zening Fu, Vince D. Calhoun, Sergey M. Plis

**Affiliations:** 1 Tri-institutional Center for Translational Research in Neuroimaging and Data Science (TReNDS), Georgia State University, Georgia Institute of Technology, Emory University, Atlanta, GA USA; 2 Department of Computer Science, Georgia State University, Atlanta, GA USA; 3 School of Computational Science and Engineering, Georgia Institute of Technology, Atlanta, GA USA; 4 Athinoula A. Martinos Center for Biomedical Imaging, Massachusetts General Hospital and Harvard Medical School, Boston, MA USA; 5 School of Electrical and Computer Engineering, Georgia Institute of Technology, Atlanta, GA USA

**Keywords:** Dynamical systems, Computer science, Alzheimer's disease, Autism spectrum disorders, Schizophrenia

## Abstract

Brain dynamics are highly complex and yet hold the key to understanding brain function and dysfunction. The dynamics captured by resting-state functional magnetic resonance imaging data are noisy, high-dimensional, and not readily interpretable. The typical approach of reducing this data to low-dimensional features and focusing on the most predictive features comes with strong assumptions and can miss essential aspects of the underlying dynamics. In contrast, introspection of discriminatively trained deep learning models may uncover disorder-relevant elements of the signal at the level of individual time points and spatial locations. Yet, the difficulty of reliable training on high-dimensional low sample size datasets and the unclear relevance of the resulting predictive markers prevent the widespread use of deep learning in functional neuroimaging. In this work, we introduce a deep learning framework to learn from high-dimensional dynamical data while maintaining stable, ecologically valid interpretations. Results successfully demonstrate that the proposed framework enables learning the dynamics of resting-state fMRI directly from small data and capturing compact, stable interpretations of features predictive of function and dysfunction.

## Introduction

Brain dynamics likely holds the key to understanding function and disorder^1–3^. The brain function manifests in a spatiotemporally localized activity within the dynamics^4^. Thus, identification and interpretation of subject-specific spatial and temporal activity may help guide our understanding of the disorder. Although, the spatiotemporal snapshots of brain dynamics can be captured noninvasively using functional magnetic resonance imaging (fMRI)^5,6^, the excessive dimensionality and complexity of fMRI signals rule out manual identification and interpretation. Alternatively, machine learning models trained to classify a mental disorder from the available observations have learned which aspects of the data reliably lead to correct prediction. In other words, the model builds internal representations of the mapping between the data and the class. Interpreting these representations can lead to discovery of previously unknown spatiotemporal functional indicators (or biomarkers).

However, standard machine learning (SML) models, when dealing directly with high-dimensional multivariate signals, suffer a drastic drop in performance because of the curse of dimensionality^7^ (high dimensionality of fMRI relative to the typically available few samples). This is because the models are usually shallow and only learn simple relationships between input and output. To improve discriminative performance, neuroimaging researchers heavily rely on measures, such as cortical thickness or connectivity matrices^8,9^, that summarize spatiotemporal relationship between different brain regions^10,11^. They apply some feature selection procedure on top of these measures to extract potentially useful features^12^ to feed into the SML model. Arguably, such proxy, bias-prone representations rely on strict assumptions and miss the chance to discover highly predictive holistic representations of the underlying dynamics^13,14^. Moreover, non-linear SML models are not easily interpretable.

Deep learning (DL) methods, on the other hand, are capable of learning complex hierarchical representations directly from the raw data through an increasingly higher level of abstraction. Recently, a large number of studies^8,15–17^ reported deep learning (DL) models’ potential in neuroimaging domains. For example, Abrol et al. (2021)^18^ demonstrated the advantages of DL models trained on raw data over SML models trained on pre-engineered features in structural magnetic resonance imaging (sMRI). The study also suggests that the deep representations of dynamics (fMRI) may be as discriminative and informative as their structural counterparts (sMRI). This automatic extraction of features with minimal guidance may greatly facilitate discovering actionable causal knowledge about the disorder by leveraging robust model introspection techniques. Model introspection, also called model interpretation, extracts the rationale behind a model’s prediction via post hoc feature attribution. This feature attribution results in a saliency map for every prediction and represents the spatiotemporal activity indicative of the disorder. However, we need to carefully design a model architecture because not every DL model is simultaneously predictive and interpretable for time series data capturing dynamics^19^.

The predictive performance of a DL model is strongly proportional to the size of training data^20,21^, which in most neuroimaging studies is scarce to come by due to the costly data collection process. In such a scenario, transfer learning can be a convenient approach to dealing with this problem, as reported in numerous studies^22–26^. Although transfer learning usually involves supervised pretraining of a model on a related task, it is difficult to find a way to formulate the pretraining task and also the data to use so as to benefit the downstream fMRI tasks. Model interpretation may be challenging for overparameterized models, but if the architecture supports robust and stable sensitivity analyses^27,28^, the interpretations for individual predictions will also be stable and robust.

**Figure 1:**
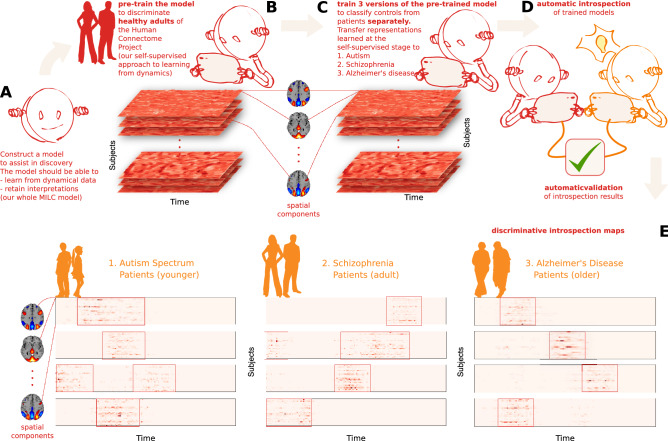
An overview of our approach to model interpretation (created in program Inkscape 1.1.2, http://inkscape.org/release/inkscape-1.1.2). (**A**) Construct a model for disorder-specific discovery: we divided the entire ICA time courses into multiple sliding windows. Then we fed them into the *whole* MILC model that learns directly from the disorder signal dynamics and retains interpretations for further introspection. (**B**) Leverage self-supervised pretraining to distinguish healthy subjects: learned representations assist the model in maintaining its predictive power when downstream training data is limited. (**C**) Construct a downstream model to discriminate patients from controls for each disorder starting with the pre-trained *whole* MILC weights: transfer of representations learned during pretraining simplifies convergence and balances overfitting. (**D**) Introspection of the trained downstream models: we compute saliency maps as a rationale used by the model behind every prediction using interpretability methods to extract meaningful, distinctive parts of the data. Subsequently, the estimated salient aspects of the dynamics go through an automatic validation process. To this end, we use the most salient features to retrain an independent SML model that confirms the salience of the features. This information can then be relayed to a human expert in the relevant field to interpret further and advance knowledge about the disorders. (**E**) Examples of saliency maps as deemed highly predictive by the models for their predictions in three different discriminative tasks. Please note that the red boxes mark the highly discriminative salient parts of the data.

The main idea of this paper is that DL can learn directly from high-dimensional signal dynamics even in small datasets and, upon introspection, can help discover disease-specific salient data regions, which, if carefully utilized, can advance our understanding of brain function. To achieve this, we introduce a model that learns from dynamical data and lends itself to interpretations. To maximally benefit from small data, we propose a self-supervised pretraining scheme^24,25^, which maximises “mutual information local to (whole) context” *whole* MILC, to capture potentially valuable knowledge from the data not directly related to the study. We use the keyword “*whole*” to emphasize that the self-supervised pretraining relies on the idea of mutual information maximization between the whole sequence (context embedding) and local windows (local embedding) from the same sequence. Our pretraining leverages publicly available healthy control subjects from the Human Connectome Project (HCP)^29^ to establish prior knowledge about the general signal dynamics and directly transfer the insights into the downstream small data studies of schizophrenia, autism, and Alzheimer’s disease with subject age-range significantly broader than in HCP. We also propose a “**R**etain **A**nd **R**etrain” (RAR) method to validate that the biomarkers identified as explanations behind the model’s predictions capture the essence of the disorder-specific brain dynamics. A visual depiction^30^ of the proposed framework is shown in Fig. 1.

## Results

We first describe all the datasets and present the results under two broad sections—*whole* MILC * Performance* and *Post hoc Explanation &* RAR * Evaluation on FNC*. The *whole* MILC * performance* indicates its predictive capacity in discriminating patients from healthy controls for each disorder separately. *Post hoc explanations* are feature attributions as determined by the *whole* MILC model for its predictions which we subsequently evaluated using the RAR scheme via an independent SVM model.

### Datasets

We used the Autism Brain Imaging Data Exchange (ABIDE)^31^(569 subjects- 255 healthy controls (HC) and 314 patients) for autism spectrum disorder (ASD), the Function Biomedical Informatics Research Network (FBIRN)^32^ (311 subjects- 151 healthy controls and 160 patients) for schizophrenia (SZ), and the Open Access Series of Imaging Studies (OASIS)^33^(372 subjects- 186 healthy controls and 186 patients) for Alzheimer’s disease (AZ).

### whole MILC performance

We evaluated the effectiveness of the proposed DL architecture with (w/) and without (w/o) the proposed self-supervised pretraining scheme, aka *whole* MILC, by comparing its performance against standard machine learning models. We also progressively increased the downstream sample size to investigate its impact on the model’s discriminative capacity. We used a K-fold cross-validation strategy for all the experiments below. The model was trained on samples progressively selected from the train folds, and we report the performance (AUC) on the test fold.

#### whole MILC evaluation

##### Autism (ABIDE)

**Figure 2:**
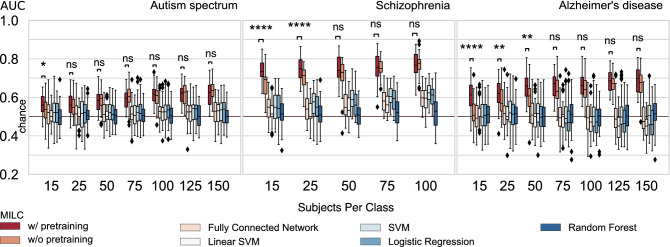
The main results from the *whole* MILC architecture and its comparison with standard machine learning models (SML). Apparently, the *whole* MILC model, in general, can learn from the raw data where traditional SML models fail to maintain their predictive capacity. Moreover, the *whole* MILC w/ pretraining substantially improves the latent representations as reflected in the improved accuracy compared to the *whole* MILC w/o pretraining. Specifically, in most small data cases, the *whole* MILC w/ pretraining outperformed the *whole* MILC w/o pretraining across the datasets. However, as expected, when we gradually increased the number of subjects during training, the effect of pretraining on the classification performance diminished, and both configurations of *whole* MILC did equally well. We verified this trend over three datasets that correspond to autism spectrum disorder, schizophrenia, and Alzheimer’s disease. Please note the Wilcoxon rank test results between w/ and w/o pretraining performance of the model as marked by asterisk (*) and “ns” (not significant), where $$ns: p > 5e^{-2}$$, $$*: 1e^{-2} < p \le 5e^{-2}$$, $$**: 1e^{-3} < p \le 1e^{-2}$$, $$***: 1e^{-4} < p \le 1e^{-3}$$, $$****: p \le 1e^{-4}$$.

Results (with K = 5) (see Fig. 2 Autism spectrum panel) show that when we used a small number of subjects for training (e.g., 15 subjects per class), the pretraining improved the model’s performance compared to when the model learned only from the downstream training data (“w/o pretraining”). However, as we gradually increased the training samples, the model w/ and w/o pretraining performs almost equally. The statistical significance test results as shown in Fig. 2 further justify our observations. The reduced effects of pretraining on autism disorder classification are reasonable because the subjects from the HCP dataset are from different age groups than those from the ABIDE dataset.

##### Schizophrenia (FBIRN)

Results (with K = 5) (see Fig. 2 Schizophrenia panel) show that the proposed architecture w/ pretraining outperformed w/o pretraining at almost all sample sizes, and the difference was more pronounced at smaller sample sizes.

##### Alzheimer’s disease (OASIS)

Similar to what has been observed in the case of SZ (FBIRN), the effect of pretraining on the downstream classification task (K = 6, to keep the testing size similar to Schizophrenia) (see Fig. 2 Alzheimer’s disease panel) was more pronounced (comfortably outperforming) than w/o pretraining. This margin was substantial when the training data size was limited. However, as we increased the training data size, the gap between “w/ pretraining” and “w/o pretraining” was hardly conceivable.

### Post hoc explanation & RAR evaluation using FNC

Once the *whole* MILC model was trained, we computed the feature attributions (saliency maps) as determined by the model for each prediction using model introspection techniques. These feature attribution values were estimated for every subject from the dataset because the subsequent validation depends on training and test samples. We used the RAR technique and an independent SVM classifier to validate the high discriminative power of the salient parts of data as identified by the model. Before RAR evaluation, we computed the average importance values of the overlapped time steps to obtain a single attribution value for every spatiotemporal dimension in the input sample. Refer to Fig. 1 for example introspection maps (saliency maps) of patients from all the relevant disorder datasets.

#### RAR evaluation

**Figure 3:**
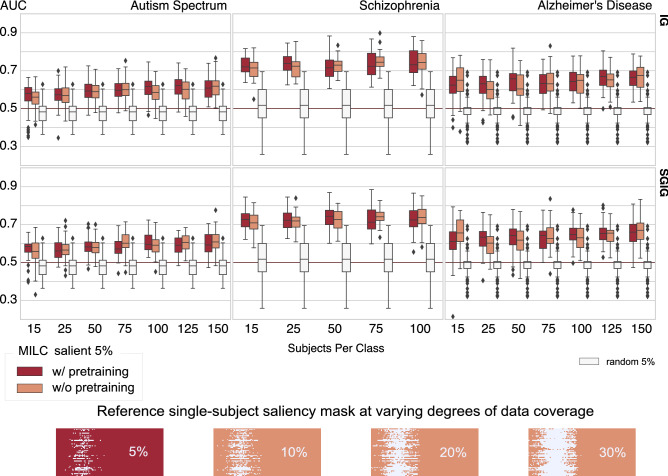
RAR employs SVM to classify the FNCs of the top 5% of the salient input data as estimated by the *whole* MILC model’s predictions. We used integrated gradients (IG) and smoothgrad integrated gradients (SGIG) to compute feature attributions. It is evident that when an independent classifier (SVM) learned on every subject’s most salient 5% data, the predictive power was significantly higher compared to the same SVM model trained on the randomly chosen same amount of data. In other words, the poor performance with randomly selected data parts indicates that other parts of the data were not exclusively discriminative as the *whole* MILC estimated salient 5% data parts. We also notice that sample masks over a different percentage of data coverage gradually obscured the localization of the discriminative activity within the data. Though the SVM model gradually became predictive with increased randomly selected data coverage, which we show in Supplementary Information, this performance upgrade was due to the gradual improvement in functional connectivity estimation and not attributable to the disease-specific localized parts within the data. For every disorder (Autism spectrum disorder, Schizophrenia, and Alzheimer’s disease), the higher AUC at this 5% indicates stronger relevance of the salient data parts to the underlying disorders. Furthermore, the RAR results reflect that in most cases, when *whole* MILC was trained with limited data, the w/ pretraining models estimated feature attributions more accurately than the models w/o pretraining.

For RAR evaluation, we trained an SVM model on FNC matrices measured as Pearson’s correlations between time courses of the components obtained by spatial independent component analysis (ICA)^34^ (discussed in “Methods” section). We estimated this FNC based on only 5% salient or random (baseline) data. The RAR validation results of different models trained on three datasets with the most salient 5% (see Supplementary Fig. 1 for results from different percentages of salient data) training data are reported in Fig. 3. As we can see, the dynamics learned by the *whole* MILC model were essential to maintain its predictive capacity. We observed that the model-specified salient data parts were more predictive than a similar amount of randomly chosen input data when we evaluated them for the same classification task using an independent SVM. This encouraging performance based on the salient data implies that the model can capture spatiotemporally meaningful markers suitable for patient-control distinction. Moreover, in many cases, the biomarkers identified with the “w/ pretraining” variant of the *whole* MILC model were more discriminative than the biomarkers specified with the “w/o pretraining” version, as reflected in the SVM’s classification performance. This encouraging result generalized across the datasets, even when we used very few subjects (15) for training.

**Figure 4:**
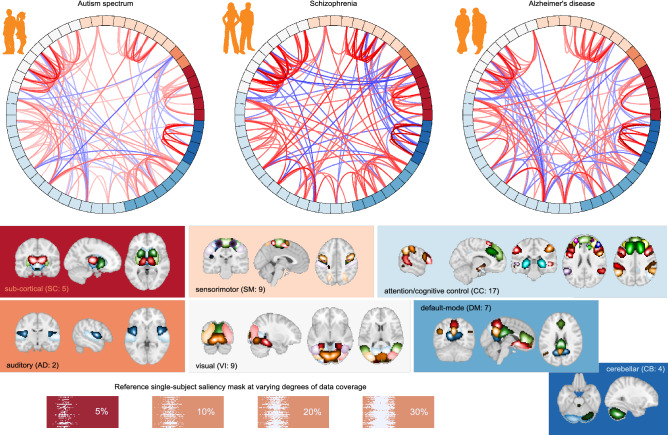
Top 10% FNC for patients computed using most 5% of the salient data as thresholded using feature attribution maps (saliency maps) for different disorders (created in programs MNE 1.1.dev0, https://mne.tools/dev/ and Inkscape 1.1.2, http://inkscape.org/release/inkscape-1.1.2). Apart from the high predictive capacity of the salient data, we observed some intriguing differences among these connectograms. The autism spectrum disorder exhibits the lowest between-domain FNC. However, salient data in autism disorder highlights domain changes in specific cerebellum, sensorimotor, and subcortical domains. The model-identified salient data reflects the most widespread pattern for schizophrenia and is consistent with the literature showing cerebellum interaction across multiple domains and sensorimotor changes. The predictive features for Alzheimer’s disease mainly concentrate on visual and cognitive interactions.

**Figure 5:**
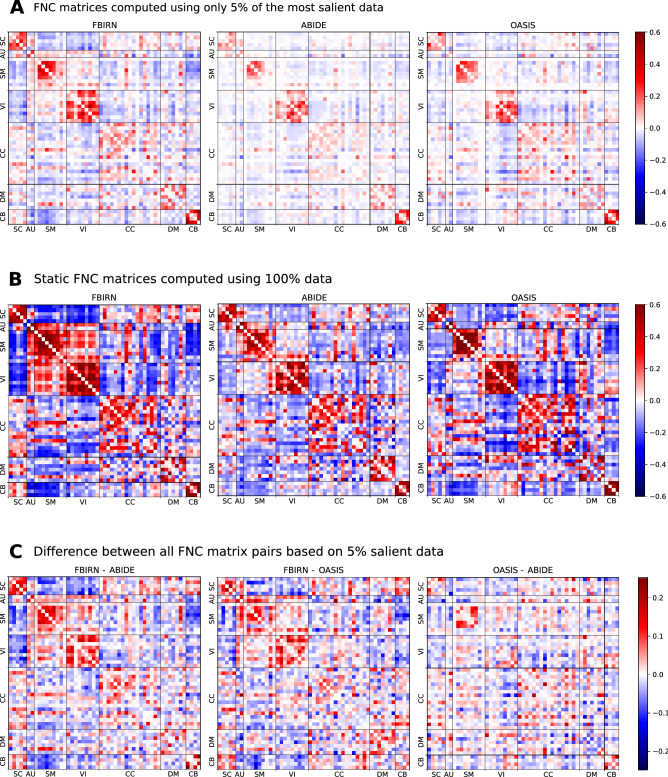
(**A**) Full FNC for patients computed using most 5% of the salient data selected based on feature attribution values for different disorders. (**B**) Static FNC (i.e., using 100% data) matrices for patients of different disorders. The FNC based on 5% salient data (**A**) does indeed convey the same focused dynamic information as currently assessed in FNC matrices based on 100% data (**B**). It is thus apparent that the proposed model can capture the focused information aligned with the current domain knowledge. (**C**) Pairwise difference of FNC matrices based on 5% salient data. The difference FNC matrices based on focused data indicate that each disorder has a uniquely distinguishable association with brain dynamics.

As demonstrated in classification performance shown in Fig. 2 and validation of feature attributions shown in Fig. 3, it is evident that the three predictive tasks were successful using our transfer learning model. In addition to quantitative validation of the automatic model introspection, we further analyzed the group-level functional network connectivity based on the model-identified salient parts of data. Refer to the connectograms (see Fig. 4) showing the top 10% FNC computed using the most 5% discriminative data as localized by the trained model for the patients in three different disorders. We can see some interesting differences in the connectograms. Autism spectrum disorder (ABIDE) shows the least between-domain FNC highlighting within domain changes in specific cerebellum, sensorimotor, and subcortical domains^35^. Schizophrenia (FBIRN) has the most widespread predictive pattern, consistent with prior work^36^ showing cerebellum interaction across multiple domains and sensorimotor changes. Finally, the predictive features for Alzheimer’s disease (OASIS) are mainly engaging visual and cognitive interactions^37^. Figure 5 shows full FNC matrices (based on 5% data), their disorder pairwise difference, and static FNC matrices (based on 100% data) for all disorders. As we can observe, the proposed model could capture the essential dynamics as generally captured in traditional full data FNC matrices and thus fully consistent with the knowledge from existing literature. The pairwise difference matrices imply that the different brain dynamics are indeed different for different disorders.

**Figure 6:**
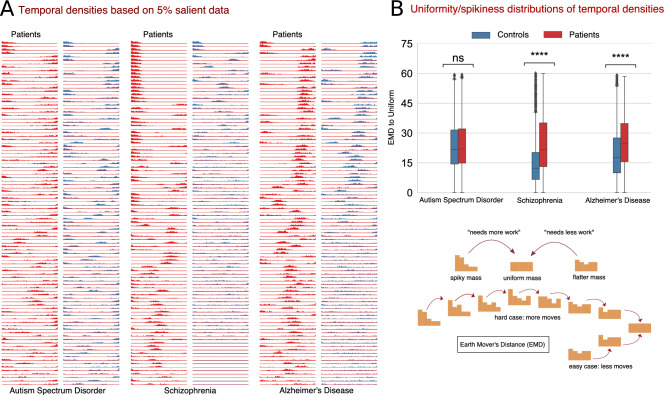
(**A**) Examples of the temporal density based on the top 5% values of the saliency maps from patients and controls for each disorder. It is noticeable that the temporal density for schizophrenia and Alzheimer’s patients is more focal in time as reflected in the spikiness, indicating that the discriminative activity for patients occurs predominantly in a shorter time interval. In contrast, for controls, model predictions do not relate to specific time intervals. For autism spectrum disorder, however, the *whole* MILC model did not capture any temporal adherence to the discriminative activity for patients. That is, the discriminatory events are not focal on shorter time intervals for ASD. (**B**) The EMD (Earth Mover’s Distance) distributions as a proxy measure for uniformity/spikiness of temporal densities (edited in program Inkscape 0.92.2, http://inkscape.org/release/0.92.2/). We analyzed the EMD measures of patients and controls to investigate the discriminative properties of salient data in terms of the spikiness or uniformity of the temporal densities. The larger EMD measures for schizophrenia and Alzheimer’s patients substantiate that the model found the discriminative activity in shorter focused time intervals. In contrast, for ASD, the equal EMD values for both patients and controls indicate that the temporal density measures do not relate to the discriminative activity for this disorder. We verified these observations with the statistical significance (Wilcoxon rank) test results as marked by asterisk (*) and “ns” (not significant), where $$ns: p > 5e^{-2}$$, $$****: p \le 1e^{-4}$$.

Furthermore, we also investigated the temporal characteristics of the saliency maps for patients and controls of each disorder. For this, we first determined the most important time points for each saliency map, expressed as temporal density and computed as the number of components for each time point that appeared in the top 5% values of the map. We observed interesting differences between groups in temporal behavior. In particular, we noticed that the temporal behavior of the most discriminative time steps is much more focused for schizophrenia and Alzheimer’s patients than their healthy controls counterparts. Put another way, the temporal density of schizophrenia and Alzheimer’s patients is generally spiky, whereas, for the healthy controls it is largely flatter. However, for autism spectrum disorder, the temporal density behavior between patients and controls is largely uniform, and the distinction, if any, is hardly noticeable. Refer to Fig. 6A for some samples showing temporal behavior of patients and controls for all disorders. To quantify these temporal characteristics (spikiness and uniformity in temporal densities), we calculated the earth mover’s distance (EMD)^38^—a distance measure between two densities—between the temporal density computed from each saliency map and a uniform density function. The intuition behind this spread measure is that a small EMD indicates that the distribution is predominantly uniform and not localized in time, implying that the discriminatory activity is usually not confined to any specific time interval. On the other side, a large EMD indicates spikiness of the temporal behavior signaling that the discriminative activity is more focused in a shorter time interval. Refer to Fig. 6B for the distributions of EMD and corresponding statistical test results for all the disorders. We observe that the discriminative activity for schizophrenia patients is predominantly local and hence more focused in time, whereas the distinguishing characteristics of healthy controls are spread across time. We observed similar characteristics for Alzheimer’s patients. However, for autism spectrum disorder, we noticed that the temporal characteristics for both patients and controls are generally spread across time and not distinguishable. We verified our observations through a non-parametric statistical test conducted on EMD distributions for each disorder.

## Discussion

Standard machine learning models are widely used in neuroimaging research partly due to their familiarity and ease of use and the perceived simplicity of interpretability of the outcomes. However, this ease/simplicity takes a hit when the complexity and dimensionality of the input data are high, as is often the case with fMRI data. Our experiments (Fig. 2) show that SML models fail to achieve good predictive performance, let alone provide meaningful interpretations of the underlying dynamics. This failure is not surprising since these proxy features are sensitive to strict assumptions about the signal dynamics^13,14^, which may only be partially accurate or accurate just under certain conditions. However, deep learning models can overcome this curse of dimensionality and learn meaningful interpretations in addition to showing high predictive performance^16–18^. This work demonstrates that DL models can achieve a deeper understanding of the underlying subject-specific signal dynamics in an fMRI setting despite the commonly expected difficulty of interpretability.

While recent advances in deep learning have proved its impressive ability to learn from a signal close to the raw data, different network architectures have benefits and limitations. The default choice of deep learning architecture for time-series data is the well-known recurrent neural network (RNN) class of models, specifically Long short-term memory (LSTM)^39^. Although LSTM models return good performance, they still have issues with interpretability due to vanishing saliency, making them unsuitable for studying multivariate signal dynamics. This necessitates building a suitable architecture that can resolve the vanishing saliency problem in the recurrent model while preserving the stability and making attributions meaningful. To that end, Ismail, Gunady, Bravo and Feizi (2020)^19^ reported that several recurrent architectures failed to provide useful attributions for the time-series data. They further reported that some architectures could extract meaningful time steps but fail to identify noteworthy features within those time steps. In this regard, we also investigated a combined CNN-RNN model and achieved high predictive performance. However, we did not find the model interpretable for time-series data. Instead, we found multi-level hierarchical attention on top of LSTM as used in *whole* MILC useful for interpretable time-series prediction. Results show that our *whole* MILC model resolves the vanishing saliency problem and is a good tool for introspection of the multivariate signal dynamics.

Interpretation of deep learning models may uncover domain-specific knowledge^40,41^ that would otherwise require high cost, effort, and time investments. Often, it may also assist in identifying if the model has inherited any inherent bias from the data. On the other hand, some studies^42,43^ raised doubts about the transparency of deep learning models and the applicability of popular interpretability methods. Notwithstanding these diverging opinions, the significance of interpretability and visualization in medicine and healthcare cannot be overstated^44^ and should involve medical experts as well. Expert human involvement in interpreting the extracted information on clinical terms may help validate and guide disease-associated discovery. A recent review^45^ reveals that deep learning models are a viable clinical supportive tool in the neuroimaging domain. However, studies have concentrated mainly on structural imaging data. Conversely, this paper introspects deep learning models for multivariate time-series data, which we think is an essential step toward interpretability research of functional imaging data. To this end, our model introspection results reveal the capacity of the proposed model to locate highly predictive disease-relevant information. Specifically, we validate the efficacy of the estimated feature attributions by proposing a method called RAR. With RAR and an independent SML model, we verify that IG and SGIG, when applied to *whole* MILC model, are robust, stable, and can demonstrably identify disorder-relevant parts of the brain dynamics. Precisely, the model-identified features offer very high predictive performance compared to random baselines for schizophrenia, Alzheimer’s disease, and autism spectrum disorders. Moreover, our FNC analysis on model introspection results, as shown in Fig. 5, harmonizes with the prior work^35–37^ for all the disorders.

We analyzed the required “what” and “when” aspects of the discriminative dynamics the model captured for patient-control distinction. Toward this goal, FNC analysis on the salient data revealed the minimally required connectivity (“what”) of the discriminative dynamics that the model used to distinguish patients from controls. We further investigated if the model leveraged any temporal (“when”) information for its discriminating power. Accordingly, we analyzed when, if such information exists, the discriminative events happen and how this temporal behavior changes between patients and controls for each disorder. As such, we analyzed the temporal densities computed from salient 5% data. Interestingly, for schizophrenia and Alzheimer’s disorders, we observed that the model used temporally dense information to distinguish patients from controls. However, no temporal association is noticed in the model behavior to distinguish ASD patients from controls. We substantiate this aspect of temporal association using a non-parametric statistical test as shown in Fig. 6.

Deep learning models typically require large amounts of data for efficient training. However, in the field of neuroimaging, collecting massive amounts of homogeneous data is infeasible thus constraining researchers to work with small data. In such cases, transfer learning^22–25^ is practically helpful to enable learning directly from data. Self-supervised learning has made significant progress in computer vision classification tasks^26^ and is equally applicable to deep convolutional and recurrent networks. As demonstrated, our self-supervised pretraining scheme^24^ enables downstream learning with minimal training data, making the direct investigation of system dynamics feasible. Our findings demonstrate that self-supervised pretraining on healthy adults dataset noticeably uplifts the downstream model’s performance on a disparate disorder dataset. These benefits generalize across datasets and disorders and thus alleviate the need to collect a massive amount of expensive data.

While the proposed framework is a stepping stone toward the direct study of signals, the proposed approach still needs to be improved to make it a clinically relevant. An interpretable model is essential to grasp better the difficult task of interpreting brain dynamics of mental disorders, and our approach demonstrably works quite well and provides a promising utility. However, a possible drawback of this current work is that the classification performance in some cases may be suboptimal due to learning directly from the signals with minimal guidance. Moreover, the spatial maps have been left unexplored. That is, utilizing only the time courses could slightly bias our models to pay more attention to the temporal component of the signal. In the future, we would like to scale our models to be able to handle full brain raw fMRI data without ICA pre-processing. We hope our interpretability approach will become even more informative in that case.

We find that interpreting DL models trained on fMRI signals to discriminate mental disorders from controls provides means to identify salient parts of brain dynamics. In particular, we show that one can capture sparse spatio-temporal signatures that encode information comparable with what is found via the traditional full data functional network connectivity analysis. We further demonstrate that the brain function manifests itself via unique dynamic signatures across time scales (latent temporality) in various disorders. Subsequently, we present an adaptive, interpretable methodology to capture these temporally transient dynamic signatures that can help distinguish disorders. Understanding the spatial and temporal specificity of the brain activity patterns will help establish the technique for clinical use by relating the differences in signature to symptoms. Moreover, to achieve these desirable disorder-specific insights, the proposed pretraining method waives the need for well-defined ground truth (biomarkers) about the disorder under consideration and a larger sample size. In the future, this method could be a significant step towards establishing more robust correlates of function-structure dependency in the brain and can also be applied more broadly to understand inter-and intraindividual variability and alterations across psychiatric disorders.

## Methods

The proposed methodology consists of 4 steps: model pretraining, downstream classification, feature importance estimation, and feature evaluation. First, we pre-trained the proposed network (*whole* MILC)^24^ on a large unrelated and unlabeled dataset to learn valuable latent representations. This pretraining, as described in the *whole* MILC section, intuitively lets the network learn foundational knowledge about the dynamics only from the healthy subjects. For pretraining and downstream tasks, we used the same model as used in^24^. However, for the current study, we replaced the CNN encoder with a recurrent encoder because we found it more stable for post hoc explanations of multivariate time-series data while interpreting the model’s predictions. As the learned dynamics are directly transferable, we used the pre-trained network to discriminate patients from healthy controls in different downstream tasks. In the second step, we trained the downstream classification model to learn more from the downstream training data dynamics. In the third step, we estimated feature importance values based on the model’s predictions using different interpretability methods (see “Model interpretability” section). In the fourth step, we evaluated the estimated features using RAR method and an SVM model as described in the RAR Section. Before going through the methodological pipeline, we preprocessed the data as described below. We state that the study was performed according to all relevant guidelines and regulations. While the original data were collected under approved IRB protocols by the original study teams, we were not involved in this step. The data were provided to us as anonymous. We submitted the proposed work to the GSU IRB which designated the project as ‘not human subjects’ thus there was no need for ongoing IRB oversight of the project.

### Preprocessing

We preprocessed the raw resting-state fMRI data using statistical parametric mapping (SPM12, http://www.fil.ion.ucl.ac.uk/spm/) package in MATLAB 2016 environment. We removed the first five scans for the signal equilibrium and participants’ adaptation to the scanner’s noise. We performed rigid body motion correction using the toolbox in SPM to correct subject head motion, followed by the slice-timing correction to account for timing difference in slice acquisition. The fMRI data were subsequently warped into the standard Montreal Neurological Institute (MNI) space using an echo-planar imaging (EPI) template. We resampled the fMRI data to $3 \times 3 \times 3$ mm$^{3}$ isotropic voxels and further smoothed using a Gaussian kernel with a full width at half maximum (FWHM) $= 6$ mm. After the preprocessing, we selected subjects with head motions $\le 3^\circ$ and $\le 3$ mm in the analysis. To ensure high data quality, we performed quality control (QC) on the spatial normalization output and removed subjects with limited brain coverage^46^. We used ICA time courses as these offer a better representation of the data than anatomical or fixed atlas-based approaches^47^. For each dataset, we used ICA components derived via a fully automated approach^48^. In this framework, we performed spatial group ICA on two independent datasets with a large sample of healthy controls (human connectome project [HCP, 823 subjects after the subject selection] and genomics superstruct project [GSP, 1005 subjects after the subject selection]) to generate network templates. For each dataset, we conducted group ICA, respectively. The estimated ICs from the two datasets were then matched by comparing their corresponding group-level spatial maps. Those pairs are considered consistent and reproducible across datasets if their spatial correlation is $\ge 0.4$. We characterized a subset of these matched ICs as ICNs instead of physiological, movement-related, or imaging artifacts. Five fMRI experts carefully examined those matched ICs, and ICs with more than three votes were considered meaningful ICNs. The experts evaluated the ICs based on the expectations that ICNs should have their activation peaks in gray matter and low spatial overlap with known vascular, ventricular, motion, and other artifacts. ICNs also should have dominant low-frequency fluctuations on their corresponding time courses (TCs). We used these meaningful ICNs as network templates for further individual-level ICA analysis. We obtained 100 ICA components for each dataset using the same procedure as described in^46^. However, this study used 53 intrinsic networks (components) for all experiments because they perfectly matched the standard network templates. In pretraining, we used a sliding window of $53 \times 20$ size with stride = 10 along the time dimension to feed the ICA time courses through a parameter-shared encoder. In all downstream classification experiments, we used a similar sliding window with stride = 1.

### Whole MILC

**Figure 7:**
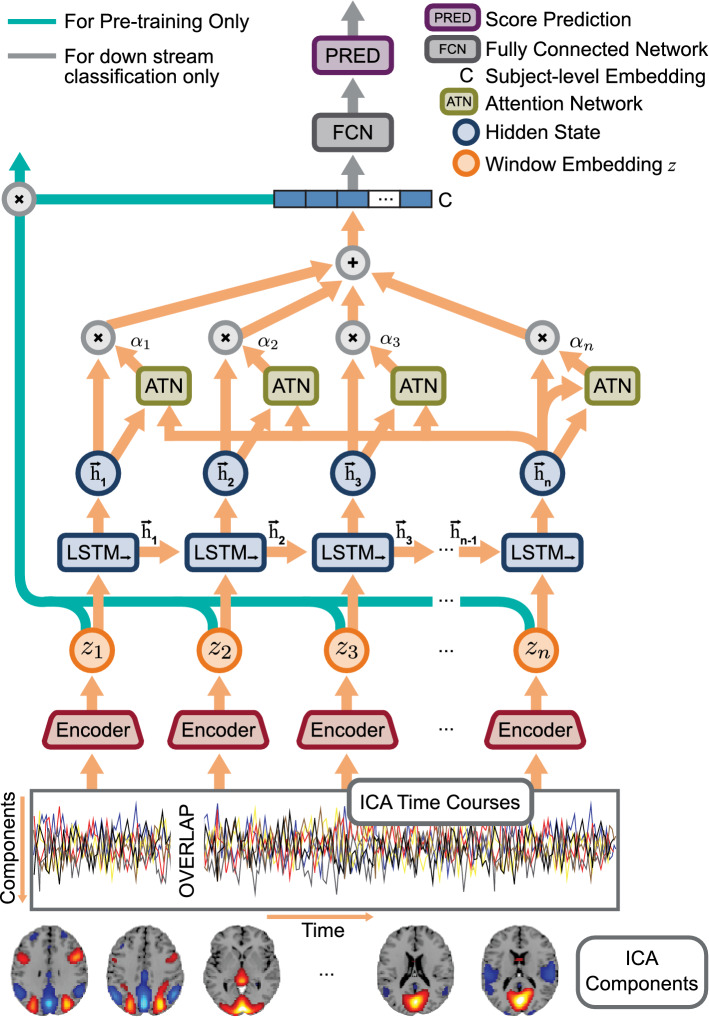
The *whole* MILC architecture—an attention-based top-down recurrent network (created in programs Adobe Illustrator 26.0.3, http://ww.adobe.com/products/illustrator.html and Inkscape 1.1.2, http://inkscape.org/release/inkscape-1.1.2). Precisely, we used an LSTM network with an attention mechanism as a parameter-shared encoder to generate the latent embeddings ${{\varvec{z}}}$ for the sliding window at all relevant positions. The top LSTM network (marked as LSTM) used these embeddings (${{\varvec{z}}}$) to obtain the global representation ${{\varvec{c}}}$ for the entire subject. During pretraining, we intended to maximize the mutual information between ${{\varvec{z}}}$ and ${{\varvec{c}}}$. In the downstream classification task, we used the global representation ${{\varvec{c}}}$ directly as input to a fully connected network for predictions. Based on these predictions, we estimated feature attributions using different interpretability methods. Finally, we evaluated the feature attributions using the RAR method and an SVM model.

The *whole* MILC model, as shown in Fig. 7, consists of two unidirectional LSTM models arranged in a top-down fashion. While the low-level LSTM functioned as a parameter-shared encoder for the sliding window over ICA time courses, the top-level LSTM used the encoder embeddings to generate a global representation for the entire sequence. Both LSTM models separately applied an attention mechanism^49^ to retain interpretable information for further model introspection. One of the benefits of the *whole* MILC model is that it is pre-trainable. Moreover, the learned representations are directly transferable to a set of downstream discriminative tasks. The *whole* MILC model used a self-supervised pretraining objective^24^ that maximized the mutual information between the latent space of a window (time slice from ICA time courses) and the corresponding whole sequence (complete ICA time courses per subject).

Let ${{{\textsf {\textit{D}}}}} = \{({\mathbf {i}}_t^{\text {i}}, \, {{\mathbf {v}}}^{\text {j}}): 1 \le {\text {t}}\le T, 1 \le {\text {i}},{\text {j}}\le N \}$ be a dataset of window-sequence embedding pairs computed from ICA time courses, where subscript ${\text {t}}$ refers to the ${\text {t}}$-th window, superscripts ${\text {i}}, {\text {j}}$ each refers to a sequence number. *T* is the number of windows in a sequence, and *N* is the total number of sequences in the dataset. ${{{\textsf {\textit{D}}}}}$ can be decomposed into a set of positive pairs ${{{\textsf {\textit{D}}}}}^+$ (${\text {i}}= {\text {j}}$) and a set of negative pairs ${{{\textsf {\textit{D}}}}}^-$ (${\text {i}}\ne {\text {j}}$) denoting a joint and a marginal distribution respectively for the window-sequence pairs in the latent space. With a separable function *f*, we used InfoNCE estimator^50^ to compute a lower bound $\mathscr {I}_f({{{\textsf {\textit{D}}}}}^+)$ on the mutual information defined as:

1 $$\begin{aligned} \mathscr {I}({{{\textsf {\textit{D}}}}}^+) \ge \mathscr {I}_f({{{\textsf {\textit{D}}}}}^+) \triangleq \sum ^N_{{\text {i}}=1} \sum ^T_{{\text {t}}=1} \log \frac{\exp f(({\mathbf {i}}_{\text {t}}^{\text {i}}, \, {{\mathbf {v}}}^{\text {i}}))}{\sum ^N_{{\text {k}}=1} \exp f(({\mathbf {i}}_{\text {t}}^{\text {i}}, \, {{\mathbf {v}}}^{\text {k}}))}, \end{aligned}$$

*f* was defined as $f({\mathbf {i}}_{\text {t}},{\mathbf {v}}) = \phi ({\mathbf {i}}_{\text {t}}^{\text {i}})^\intercal ({\mathbf {v}}^{\text {j}})$, where $\phi$ was some embedding function learnt by network parameters. *f* learned an embedding function such that it assigned higher values for positive pairs than for negative pairs, i.e., $f({{{\textsf {\textit{D}}}}}^+) \gg f({{{\textsf {\textit{D}}}}}^-)$. To make it precise, ${\mathbf {i}}_{\text {t}}$ and ${\mathbf {v}}$ in the Eq. (1) respectively refer to window embedding ${\mathbf {z}}_{{\text {t}}}$ and global sequence embedding ${\mathbf {c}}$ in Fig. 7. The InfoNCE loss using *f* as a representation model is defined as $L = - \mathscr {I}_{f}$.

#### Attention mechanism

The attention mechanism is a valuable construct commonly used in DL architecture to preserve long-term dependency in the recurrent neural network. Initially, Bahdanau, Cho, and Bengio (2014)^49^ introduced the attention mechanism for the neural machine translation to compute the relevance of source words toward each output word. However, the attention mechanism can benefit other applications too. For example, we used the attention mechanism to solve vanishing saliency problems in the LSTM networks to retain interpretable information during model training. In the attention mechanism as used in *whole* MILC model, we took all the hidden states ${\mathbf {h}}= [{\mathbf {h}}_1, {\mathbf {h}}_2, \dots , {\mathbf {h}}_n]$ from the LSTM network and concatenated each hidden state ${\mathbf {h}}_i$ with the hidden state at the last time step ${\mathbf {h}}_n$ before passing through an attention mechanism $f_a$. The attention mechanism $f_a$, similar to the additive attention mechanism introduced in^49^, took pairs of hidden states $({\mathbf {h}}_i, {\mathbf {h}}_n)$ as inputs, passed through a 2-layer feed-forward network and generated a vector of *n* alignment scores $f_a({\mathbf {h}}_i, {\mathbf {h}}_n)$. The alignment score for each time point *i* intuitively indicates the degree of relevance of the corresponding hidden state to the overall embedding. We normalized the alignment scores using $\mathrm {softmax}$ to produce a series of weights $\alpha _1, \alpha _2, \dots , \alpha _n$. $\alpha _i$ for each time point is defined as:

2 $$\begin{aligned} \alpha _i = \frac{\exp (f_a({\mathbf {h}}_i, {\mathbf {h}}_n))}{\sum ^n_{k=1} \exp (f_a({\mathbf {h}}_k, {\mathbf {h}}_n))} \end{aligned}$$

where *n* was the number of time steps over which attention was applied. Note that the value of *n* for the encoder LSTM network (for the sliding window) differed from the top LSTM network (for the full subject). The global representation ${\mathbf {c}}$ (or the window embedding ${\mathbf {z}}$) was generated using the formula as follows:

3 $$\begin{aligned} {\mathbf {c}}= \sum ^n_{k=1} \alpha _k {\mathbf {h}}_k \end{aligned}$$

#### whole MILC setup

##### Encoder embedding

The LSTM encoder with an attention mechanism used a sliding window of $53 \times 20$ size to feed the ICA time courses and encoded features at each time point into a 256-dimensional representation. At each position of the sliding window, we concatenated the hidden state for each time step $t_i$ within the window with the final hidden state of the same window as described in the attention mechanism. We then passed these concatenated 512-dimensional vectors through an attention network, a two-layer feed-forward network with hidden units 64. The network learned a series of weights representatives of the magnitude of attention regarded as important for the time steps. All the hidden representations within a window were then weighted based on the attention scales to produce window embedding ${\mathbf {z}}$.

##### Pretraining

In *whole* MILC based pretraining, we passed all the encoder embeddings ${\mathbf {z}}= {\mathbf {z}}_1, {\mathbf {z}}_2, \dots , {\mathbf {z}}_n$ to another unidirectional LSTM network with an attention mechanism. In this top recurrent network, each window embedding ${\mathbf {z}}_i$ corresponded to the input for a single time step. We used 200 dimensions to represent the hidden state for this top network. We concatenated each hidden state with the hidden state at the last time step to make it contextually relevant for the attention mechanism. The top attention network used 400 input neurons and 128 hidden units to learn *k* weights, where *k* was the number of input windows. These weights were used as coefficients in the linear combination of hidden representations to generate a global embedding ${\mathbf {c}}$ of dimension 200 for each subject. Based on ${\mathbf {c}}$ and ${\mathbf {z}}$, we pre-trained the neural network to maximize the mutual information between a window and the corresponding input sequence. We used subjects from the HCP dataset for pretraining and used 700 subjects for training and 123 subjects for the test, obtaining 89% pretraining accuracy.

##### Classification tasks

In downstream tasks, we deal with classifying subjects into patients and controls separately for each disorder. Similar to pretraining, we fed ICA time courses into the LSTM encoder using a sliding window. The LSTM encoder projected all the windows into latent representations ${\mathbf {z}}$, which were then passed to another LSTM network to obtain a global representation ${\mathbf {c}}$. Finally, on top of ${\mathbf {c}}$, we used a feed-forward network with 200 hidden units to perform binary classification. We gradually increased the number of supervised training subjects to observe the pretraining effect on downstream data size compared to the setup where we used no pretraining. For each experiment, we report cross-validated results. Moreover, we performed ten repetitions of each experimental setup, with different random seeds for every cross-validation fold to ensure stable results. For each random seed, we randomly chose the training samples as required from the available training pool.

### Model interpretability

We describe an *input* as a vector ${{\varvec{x}}}\in {\mathbb {R}}^{d}$. Let us define the deep learning model as a function $F: {\mathbb {R}}^{d} \rightarrow {\mathbb {R}}^{C}$, where *C* is the number of classes in the downstream classification problem (in our case $C=2$). Moreover, let us also assume that the mapping $F_{c} ({{\varvec{x}}}): {\mathbb {R}}^{\mathrm{d}} \rightarrow {\mathbb {R}}$ defines the class-specific logit, where *c* is the predicted class. An explanation method, also called model introspection method, provides an *explanation map* $E : {\mathbb {R}}^{d} \rightarrow {\mathbb {R}}^{d}$ that maps ${{\varvec{x}}}$ to a saliency map of the same shape. Values in the saliency map correspond to the ‘relevance’ or ‘importance’ of that dimension to the model’s prediction.

The need to enable model interpretation led to a variety of model introspection techniques that can be roughly split into three groups: (1) model-sensitive^27,28^, (2) model-agnostic^51,52^, and (3) counterfactual explanations^53^. The techniques have their relative benefits and pitfalls in addressing the desiderata of different applications^54^. Adebayo, Muelly, Liccardi, and Kim (2020)^55^ reported that, under normal conditions, gradients, smoothgrad^28^, and integrated gradients (IG)^27^ passed end-user recommendations. Additionally, the smoothgrad method^28^ resolves the problems^56^ of saliency maps, which in general, are susceptible to noise and input perturbations. Guided by these findings, we relied on IG, and smoothgrad on IG to introspect the proposed model. Notably, we found IG and smoothgrad on IG generalizable, stable, and noise-robust across the disorders.

### Random baseline

We randomly assigned feature importance values to create random baselines to validate the post hoc explanations (saliency maps). Specifically, we ordered the features uniformly at random using random permutations and considered each permutation as an order of importance. We refer to this random estimator as $g^\mathbf{R}$ throughout the paper. In contrast, we used the magnitude of the estimated attribution values as the order of importance for the model-generated post hoc explanations. To evaluate the efficacy of the estimated feature importance, we compared the predictive power of the model-estimated salient features against random baselines using a technique called RAR, which we describe below.

#### RAR method and setup

In RAR, we retained only a small percentage of the most salient features as determined by the model and replaced other features with non-informative values (zeros). We used these modified samples to retrain an SVM model to evaluate the effectiveness of the estimated feature attributions. In particular, we show that the performance obtained with *whole* MILC model-estimated salient features far exceeded the random baseline. We mathematically describe the RAR scheme as follows:

Let us define ${{{\textsf {\textit{X}}}}}$ to be the original dataset. ${{{\textsf {\textit{X}}}}}^{M} \, | \, g^{\mathbf {R}}$ be the modified dataset based on random importance estimates and ${{{\textsf {\textit{X}}}}}^M \, | \, g_i$ be the modified dataset according to the saliency maps generated by applying some interpretability method $g_i$ on *whole* MILC predictions. We computed static functional network connectivity, measured as Pearson’s correlation coefficients, for each sample in ${{{\textsf {\textit{X}}}}}^M$. We used these correlation coefficients as features to train an independent SVM model de novo. We evaluated the classification performance of the SVM models trained separately with *whole* MILC-generated salient features and randomly selected features. Indeed, we show that $\xi ({{{\textsf {\textit{X}}}}}^M \, | \, g_i) > \xi ({{{\textsf {\textit{X}}}}}^M \, | \, g^{\mathbf {R}})$, where $\xi$ is the performance evaluation function, e.g. area under the ROC curve and/or accuracy.

**Figure 8:**
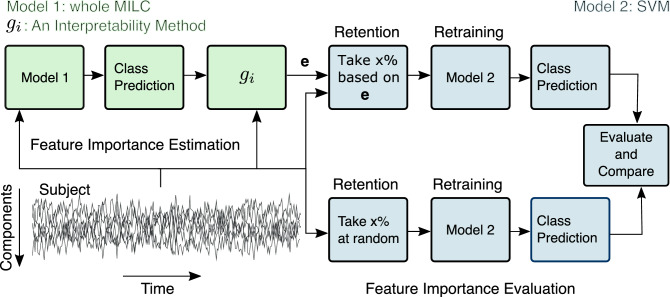
End-to-end process of RAR evaluation. For each subject in the dataset, based on the *whole* MILC class prediction and model parameters, we estimated the feature importance vector $\mathbf {{{\varvec{e}}}}$ using some interpretability method $g_i$. Later on, we validated these estimates against random feature attributions $g^{\mathbf {R}}$ using the RAR method and an SVM model. Through the SVM model’s performance when separately trained with different feature sets, we show that *whole* MILC model-estimated features were highly predictive compared to a random selection of a similar amount of features. Empirically, we show that $\xi ({{{\textsf {\textit{X}}}}}^M \, | \, g_i) > \xi ({{{\textsf {\textit{X}}}}}^M \, | \, g^{\mathbf {R}}$), where $\xi$ is the performance evaluation function (e.g., area under the curve) and ${{{\textsf {\textit{X}}}}}^M$ refers to the modified dataset constructed based on only retained feature values.

It is to note that we sorted the features based on their signed attribution values before considering them for validation. We searched for the SVM (nonlinear) parameters using a parameter grid and 3-fold cross-validation on the training data. We used the same folds and train-test splits for the RAR evaluation as used in the *whole* MILC model. Figure 8 shows the schematic of the end-to-end process: (1) training the *whole* MILC and feature attributions and (2) Evaluation of the feature attributions using RAR and an SVM model.

## Supplementary Information

Supplementary Figure 1.
