# Supplementary material for: Interpreting models interpreting brain dynamics
Source: Sci Rep. 2022 Jul 21;12:12023. doi: 10.1038/s41598-022-15539-2 (PMC9304350; doi:10.1038/s41598-022-15539-2)
Supplement: Supplementary file 1 — Supplementary Figure 1. [file 41598_2022_15539_MOESM1_ESM.docx]

Supplementary Fig. 1: RAR evaluation on different percentages of salient data

RAR employs an SVM to validate FNCs computed using different percentages of the salient input data (5% - 30%) as determined through post hoc explanations of the whole MILC model's predictions. When an independent classifier (SVM) was trained on every subject's most salient 5% - 10% data, the predictive power was significantly higher compared to the same SVM classifier trained on randomly chosen identical amounts of data. If we retain a minimal amount of data (5% - 10%), the lower performance with randomly selected data parts indicates that these (randomly chosen) data parts are not exclusively discriminative as the whole MILC estimated salient (5% - 10%) data parts. However, if we retain a higher percentage (e.g., 20% - 30%) of data coverage, the performance gap between the SVM models trained respectively with saliency-based data coverage and randomly selected data coverage diminishes. This observation is reasonable as a higher percentage of random data selection eventually captures lots of salient data parts and improves the SVM model's performance comparably. However, as our results in Figure 2 of the main paper demonstrate, SVM will again fail to predict the class if it directly learns from the complete time courses. Precisely, we show that the most salient localized data identified by the whole MILC model are indeed highly discriminative for the underlying disorder. The **whole MILC (x, x)** specifies an instance of the model trained only on **x** number of patients and **x** number of controls.
